# Cigarette smoke inhibits BAFF expression and mucosal immunoglobulin A responses in the lung during influenza virus infection

**DOI:** 10.1186/s12931-015-0201-y

**Published:** 2015-03-14

**Authors:** Jianmiao Wang, Qinghai Li, Jungang Xie, Yongjian Xu

**Affiliations:** Department of Respiratory and Critical Care Medicine, Tongji Hospital, Tongji Medical College, Huazhong University of Science and Technology, 1095 Jiefang Road, Wuhan, 430030 China

**Keywords:** B cell activating factor belonging to the tumor necrosis factor family (BAFF), Chronic obstructive pulmonary disease, Cigarette smoking, Immunoglobulin A, Influenza

## Abstract

**Background:**

It is incompletely understood how cigarette smoke (CS) exposure affects lung mucosal immune responses during viral respiratory infections. B cell activating factor belonging to the tumor necrosis factor family (BAFF) plays an important role in the induction of secretory immunoglobulin A (S-IgA) which is the main effector of the mucosal immune system. We therefore investigated the effects of CS exposure on BAFF expression and S-IgA responses in the lung during influenza virus infection.

**Methods:**

Mice were exposed to CS and/or infected with influenza virus. Bronchoalveolar lavage fluid and lung compartments were analyzed for BAFF expression, influenza-specific S-IgA level and histological changes. Lung B cells were isolated and the activation-induced cytidine deaminase (Aicda) expression was determined. BEAS-2B cells were treated with CS extract (CSE), influenza virus, interferon beta or N-acetylcysteine and BAFF expression was measured.

**Results:**

CS inhibited BAFF expression in the lung, particularly after long-term exposure. BAFF and S-IgA levels were increased during influenza virus infection. Three-month CS exposure prior to influenza virus infection resulted in reduced BAFF and S-IgA levels in the lung as well as augmented pulmonary inflammation on day 7 after infection. Prior CS exposure also caused decreased Aicda expression in lung B cells during infection. Neutralization of BAFF in the lung resulted in reduced S-IgA levels during influenza virus infection. CSE inhibited virus-mediated BAFF induction in a dose-dependent manner in BEAS-2B cells, while this inhibition of BAFF by CSE was prevented by pretreatment with the antioxidant N-acetylcysteine.

**Conclusions:**

Our findings indicate that CS may hinder early mucosal IgA responses in the lung during influenza virus infection through oxidative inhibition of BAFF, which might contribute to the increased incidence and severity of viral infections in smokers.

**Electronic supplementary material:**

The online version of this article (doi:10.1186/s12931-015-0201-y) contains supplementary material, which is available to authorized users.

## Background

Large epidemiologic studies clearly show that cigarette smoking is associated with an increased incidence of respiratory infections such as influenza, and clinical observations also suggest that these infections are more severe among smokers [1]. Moreover, chronic obstructive pulmonary disease (COPD), a condition mainly caused by cigarette smoke (CS) exposure, is associated with periods of acute exacerbation of symptoms largely due to respiratory infections [2]. These findings suggest that CS exposure may affect respiratory tract defense mechanisms against pathogens.

Respiratory mucosal immune responses are critical for protecting the lung from potentially harmful effects of infections [3]. Some studies show that CS exposure impacts many cell types of mucosal immune system including epithelial cells, macrophages, dendritic cells (DCs) and lymphocytes [4,5]. Other studies also suggest that the levels of secretory immunoglobulin A (S-IgA) are reduced in the bronchoalveolar lavage (BAL) fluid of COPD patients [6]. However, it is incompletely understood how CS exposure affects mucosal immune responses during respiratory infections.

S-IgA is the main effector of mucosal immune system and plays an important role in immune protection against infectious agents through immune exclusion, intracellular neutralization and antigen excretion [7]. IgA class switching is the process whereby B cells acquire the expression of IgA, which occurs via both T cell-dependent (TD) and T cell-independent (TI) pathways [8]. The TD pathway requires at least 5–7 days to develop, a delay that could prove fatal in the presence of mucosal infections [8]. However, IgA can be rapidly produced through the faster TI pathway involving B cell activating factor belonging to the tumor necrosis factor family (BAFF) [9,10].

BAFF is an important cytokine for B cell survival and maturation [11], which is expressed by many cell types including macrophages, DCs and epithelial cells [12]. It binds to three receptors on B cells including BAFF receptor (BAFF-R), transmembrane activator and calcium modulator cyclophilin ligand interactor (TACI) and B cell maturation antigen (BCMA) [12]. Previous studies demonstrate that BAFF and its homolog, a proliferation-inducing ligand (APRIL), can induce TI IgA class switching and activation-induced cytidine deaminase (Aicda; an enzyme essential for IgA class switching) expression in B cells [9,13]. Recent studies also show that BAFF but not APRIL expression is increased in the airway during respiratory syncytial virus (RSV) infections [14].

However, BAFF expression and IgA responses in the lung during viral infections following CS exposure have not been studied. In the present study, we utilized murine and cellular models to investigate the effects of CS exposure on BAFF expression and mucosal IgA responses during influenza virus infection.

## Methods

### Animals

C57BL/6 mice were purchased from the animal center of Tongji Medical College of Huazhong University of Science and Technology (Wuhan, China). All animal experiments were approved by the Institutional Animal Care and Use Committee of Tongji Medical College.

### CS exposure

Six to 8 weeks old male mice were exposed to room air (RA) or the smoke from nonfiltered 3R4F research cigarettes (University of Kentucky, Lexington, KY, USA) using the smoking apparatus as previously described [15]. Mice received a half cigarette twice a day to allow for acclimation in the first week and received 1 cigarette twice a day thereafter.

### Influenza virus infection

Six to 8 weeks old male mice were anesthetized and 1.5 × 10^3^ plaque forming units of A/PR8/34 (H1N1) influenza virus (Advanced Biotechnologies, Columbia, MD, USA) was administered via nasal aspiration in 50 μl of phosphate buffered saline (PBS) using techniques previously described [16].

### BAFF neutralization

For neutralization of BAFF in the lung, mice were anesthetized and intranasally administered with 10 μg of recombinant mouse BAFF-R Fc fusion protein (BAFF-R-Fc; R & D, Minneapolis, MN, USA) in 50 μl of PBS 1 day prior to influenza virus infection, followed by subsequent administration of the same dose of BAFF-R-Fc on day 1, 3 and 5 after infection. As control, mice were administered with 10 ug of isotype control (IgG1) or 50 μl of PBS alone in the same schedule as BAFF-R-Fc delivery.

### BAL

Mice were sacrificed and the trachea was cannulated and perfused with two 0.75 ml aliquots of PBS. The cellular contents and BAL fluid were separated by centrifugation. Total and differential leukocyte counts were determined and BAL fluid samples were stored at minus 80°C until analyzed.

### B cell isolation

Lung single cell suspensions were prepared using the Lung Dissociation Kit (Miltenyi Biotec, Auburn, CA, USA) according to the protocol described previously [17]. Cells were incubated with mouse B220 MicroBeads (Miltenyi Biotec) based on the protocol supplied by the manufacturer, and then positively selected for mRNA analysis.

### Preparation of CS extract

CS extract (CSE) was freshly prepared by bubbling the smoke from two 3R4F research cigarettes without filter, at a rate of 1 cigarette/5 min, to a 50 ml conical tube containing 20 ml culture medium. The extract was filtered through a 0.22 μm filter and was regarded as 100% strength CSE.

### Cell culture

BEAS-2B cells (human bronchial epithelium; American Type Culture Collection, Manassas, VA, USA) were cultured in RPMI 1640 medium supplemented with 10% fetal bovine serum and 1% penicillin/streptomycin in a humidified incubator under 5% CO_2_ at 37°C. Experiments were performed on cells at 80% confluence. Cells were treated with various concentrations of CSE and/or infected with influenza virus at a multiplicity of infection (MOI) of 0.5 for 24 hours. In the experiments of interferon beta (IFN-β) treatment, cells were treated with various concentrations of recombinant human IFN-β (PBL Assay Science, Piscataway, NJ, USA) alone or in the combination with 5% CSE and influenza virus at 0.5 MOI for 24 hours. In the experiments of N-acetylcysteine (NAC) treatment, cells were pretreated with various concentrations of NAC (Sigma-Aldrich, St. Louis, MO, USA) for 2 hours and then treated with 5% CSE and influenza virus at 0.5 MOI for 24 hours.

### Enzyme-linked immunosorbent assay

BAFF protein levels in BAL fluid were quantified using commercial enzyme-linked immunosorbent assay (ELISA) kits (R & D) according to the manufacturer’s instructions. Influenza-specific S-IgA levels in BAL fluid were determined by ELISA as described previously [18,19]. S-IgA antibody titers were defined as the reciprocal of the highest dilution of sample for which the optical density was at least twice that of negative controls. Endpoint S-IgA levels were expressed as reciprocal log_2_ titers.

### Quantitative polymerase chain reaction

Total RNA was isolated from the lung tissues, lung B cells, and BEAS-2B cells using RNeasy Plus Mini Kit (Qiagen, Valencia, CA, USA). After synthesis of cDNA, quantitative polymerase chain reaction (PCR) was performed using SsoAdvanced Universal SYBR Green Supermix (Bio-Rad, Hercules, CA, USA) and the specific primers. The primer sequences are listed in Table 1. The relative mRNA expression was determined using the 2^-ΔΔCt^ methods with the 18S rRNA as endogenous control.

**Table 1:** **Primer sequences used for quantitative polymerase chain reaction**

**Gene**	**Forward primer**	**Reverse primer**
BAFF	5′-AAACACTGCCCAACAATTCC-3′	5′-TTGCGTGAAATCTGTGCATT-3′
BAFF (human)	5′-ACCGCGGGACTGAAAATCT-3′	5′-CACGCTTATTTCTGCTGTTCTGA-3′
KC	5′-TGCACCCAAACCGAAGTCAT-3′	5′-TTGTCAGAAGCCAGCGTTCAC-3′
APRIL	5′-TCACAATGGGTCAGGTGGTATC-3′	5′-TGTAAATGAAAGACACCTGCACTGT-3′
Aicda	5′- TGCTACGTGGTGAAGAGGAG-3′	5′- TCCCAGTCTGAGATGTAGCG-3′
BAFF-R	5′-CCCCAGACACTTCAGAAGGA-3′	5′-AGGTAGGAGCTGAGGCATGA-3′
TACI	5′-GTGTGGCCACTTCTGTGAGA-3′	5′-CTGGTGCCTTCCTGAGTTGT-3′
BCMA	5′-ATCTTCTTGGGGCTGACCTT-3′	5′-CTTTGAGGCTGGTCCTTCAG-3′
M1 (viral)	5′-AAGACCAATCCTGTCACCTCTGA-3′	5′-CAAAGCGTCTACGCTGCAGTCC-3′
18S rRNA	5′-CGCGGTTCTATTTTGTTGGTTT-3′	5′-GCGCCGGTCCAAGAATTT-3′

### Histology and immunohistofluorescence

Lungs were perfused with 20 ml PBS through the right ventricle and the right main bronchus was ligated. The left lung was inflated and fixed with 10% formalin for 24 hours. The lungs were then paraffin embedded. Hematoxylin and eosin (H & E) staining was performed by standard protocols. For immunohistofluorescence staining, lung sections were dewaxed, rehydrated and subjected to heat-induced epitope retrieval. After blocking, sections were incubated with rat anti-BAFF monoclonal IgM antibody [Buffy 2] (Abcam, Cambridge, MA, USA) at 4°C overnight. After washing, sections were incubated with Alexa Fluor 594 goat anti-rat IgM (μ chain) (Life Technologies, Grand Island, NY, USA) for 1 hour at room temperature in the dark, and then washed and coverslipped with Vectashield HardSet Mounting Medium with DAPI (Vector Laboratories, Burlingame, CA, USA).

### Statistical analysis

All data were expressed as mean (± SEM) values, unless otherwise noted. The student unpaired two-tailed *t* test was performed for all statistical analyses using GraphPad Prism 6 software (GraphPad, San Diego, CA, USA). Differences between groups were considered significant when *P* < 0.05.

## Results

### CS induces inflammation but inhibits BAFF expression in the lung

**Figure 1:**
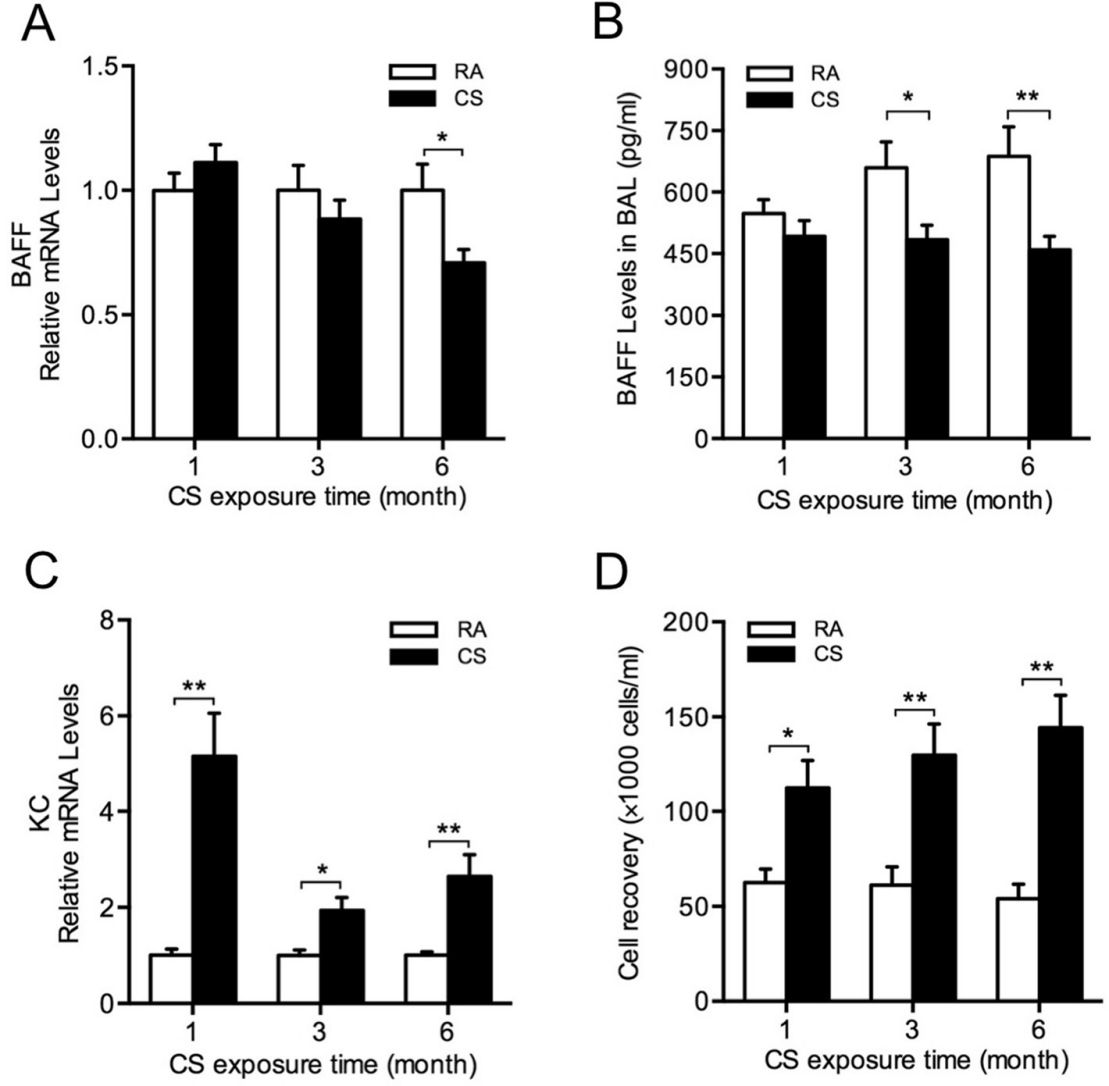
**Cigarette smoke (CS) induces inflammation but inhibits BAFF expression in the lung.** Mice were exposed to room air (RA) or CS for 1 month, 3 months or 6 months. The relative mRNA levels of **(A)** BAFF and **(C)** keratinocyte-derived chemokine (KC) in the lung, **(B)** BAFF protein levels and **(D)** the total leukocyte counts in bronchoalveolar lavage (BAL) fluid are shown (n = 5–10 mice/group). *P < 0.05; **P < 0.01.

To study BAFF expression in the lung during CS exposure, mice were exposed to RA or CS for 1 month, 3 months or 6 months. We found that short-term CS exposure did not alter BAFF expression significantly. However, BAFF mRNA expression was decreased significantly in the lung of mice exposed to CS for 6 months compared with RA-exposed controls (Figure 1A), and BAFF protein levels were reduced significantly in BAL fluid after 3-month or 6-month CS exposure (Figure 1B). In addition, the mRNA expression of keratinocyte-derived chemokine (KC; a mouse homolog of interleukin-8) in the lung and the total leukocyte counts in BAL fluid were increased significantly at all timepoints (Figure 1C and D).

### Influenza virus induces BAFF expression and mucosal IgA responses in the lung

**Figure 2:**
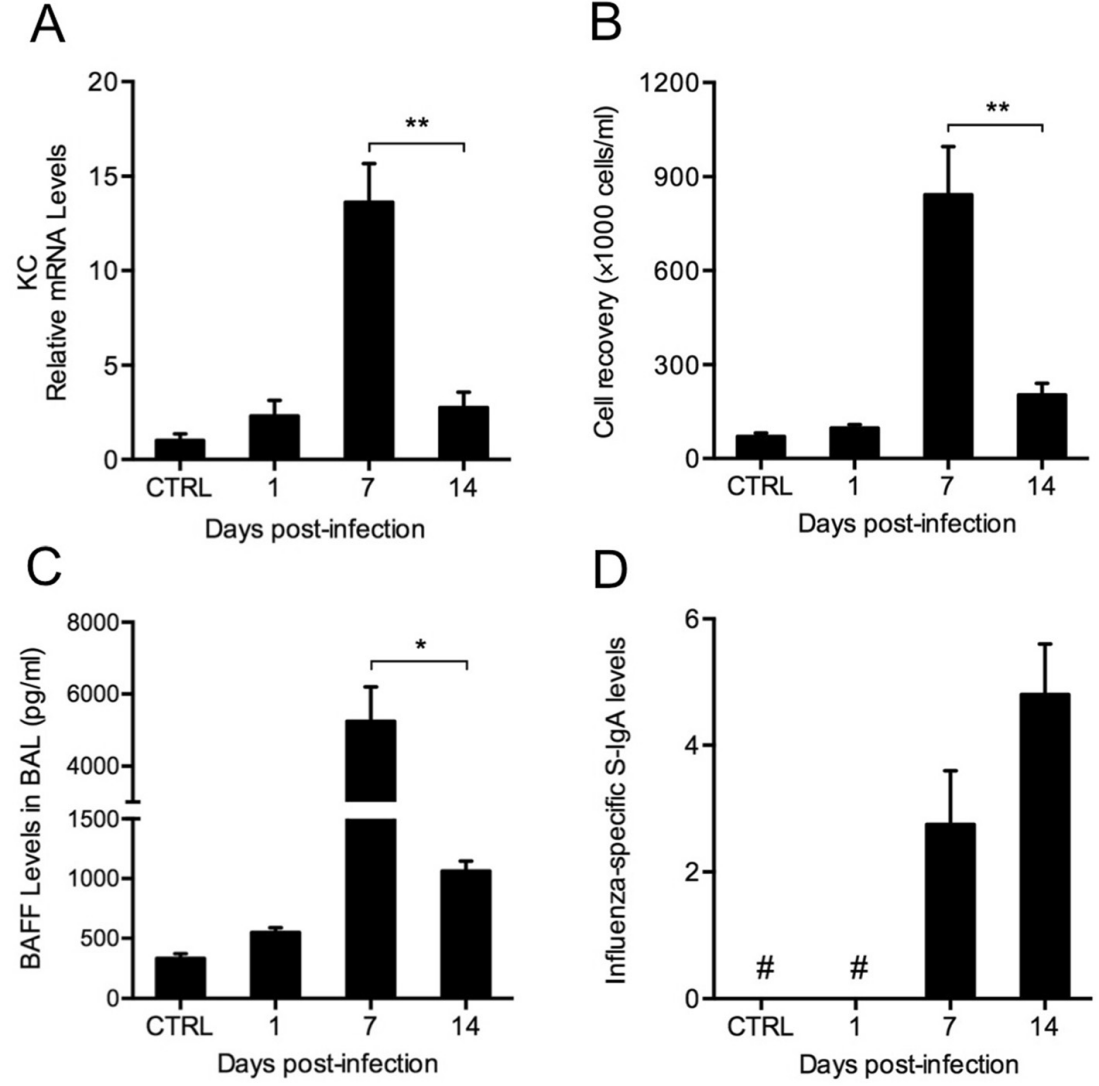
**Influenza virus induces BAFF expression and mucosal IgA responses in the lung.** Mice were infected with influenza virus and sacrificed on day 1, day 7 and day 14 after infection. **(A)** The relative mRNA levels of keratinocyte-derived chemokine (KC) in the lung, **(B)** the total leukocyte counts, **(C)** BAFF protein levels and **(D)** influenza-specific secretory IgA (S-IgA) levels in bronchoalveolar lavage (BAL) fluid are shown (n = 3–5 mice/group). CTRL, controls; #, not detected; *P < 0.05; **P < 0.01.

To investigate BAFF expression and mucosal IgA responses in the lung during influenza virus infection, mice were infected and sacrificed on day 1, 7 and 14 after infection. We found that KC in the lung and the total leukocyte counts in BAL fluid were increased during infection, and both peaked on day 7 and declined significantly on day 14 (Figure 2A and B). BAFF was induced rapidly and highly by influenza virus, which also reached a peak value on day 7 and declined on day 14 (Figure 2C). The influenza-specific S-IgA levels in BAL fluid were also increased markedly on day 7 and 14 (Figure 2D).

### CS exposure prior to influenza virus infection results in reduced BAFF and S-IgA levels as well as augmented lung inflammation

**Figure 3:**
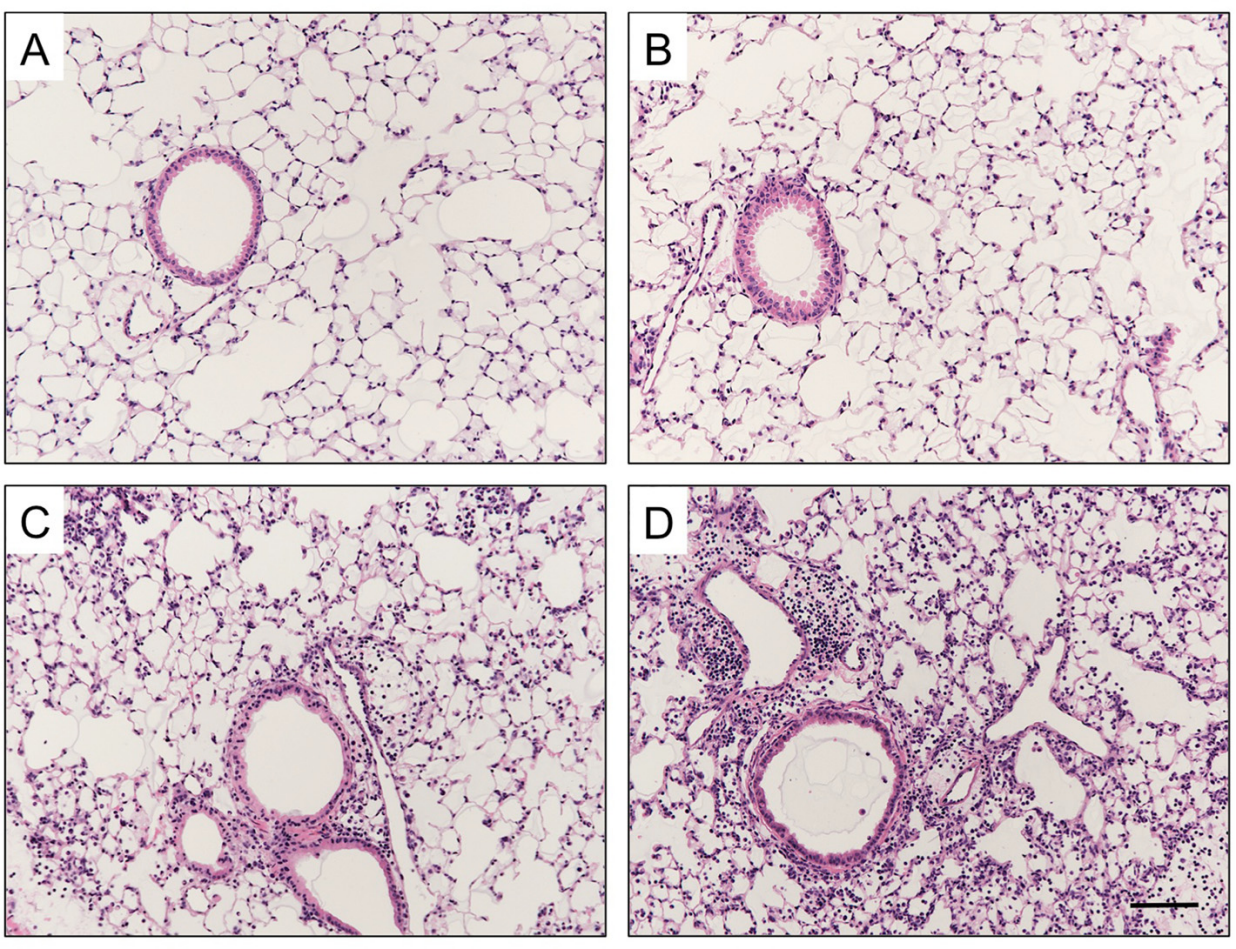
**Cigarette smoke (CS) exposure prior to influenza virus infection results in augmented lung inflammation.** Mice were exposed to room air (RA) or CS for 3 months and then infected with influenza virus or vehicle control. They were sacrificed and evaluated on day 7 after infection. Representative haematoxylin and eosin stained lung sections from **(A)** RA-exposed mice, **(B)** CS-exposed mice, **(C)** RA-exposed mice with influenza virus infection and **(D)** CS-exposed mice with influenza virus infection are shown. Scale bar, 100 μm; Original magnification, ×100.

**Figure 4:**
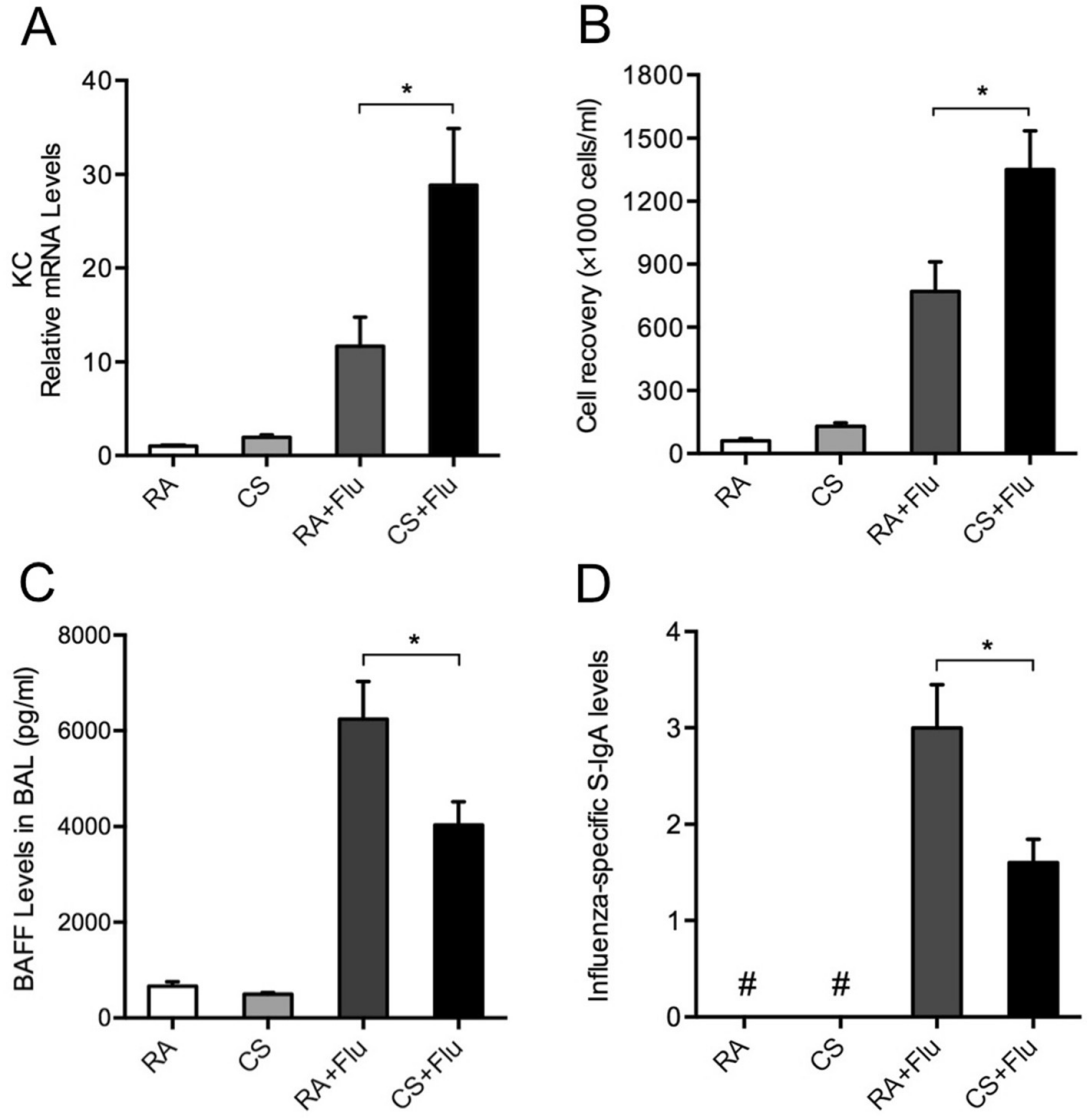
**Cigarette smoke (CS) exposure prior to influenza infection results in reduced BAFF and secretory IgA (S-IgA).** Mice were exposed to room air (RA) or CS for 3 months and then infected with influenza virus or vehicle control. They were sacrificed and evaluated on day 7 after infection. **(A)** The relative mRNA levels of keratinocyte-derived chemokine (KC) in the lung, **(B)** the total leukocyte counts, **(C)** BAFF protein levels and **(D)** influenza-specific S-IgA levels in bronchoalveolar lavage (BAL) fluid are shown (n = 5 mice/group). RA + Flu, RA-exposed mice with influenza virus infection; CS + Flu, CS-exposed mice with influenza virus infection; #, not detected; *P < 0.05.

**Figure 5:**
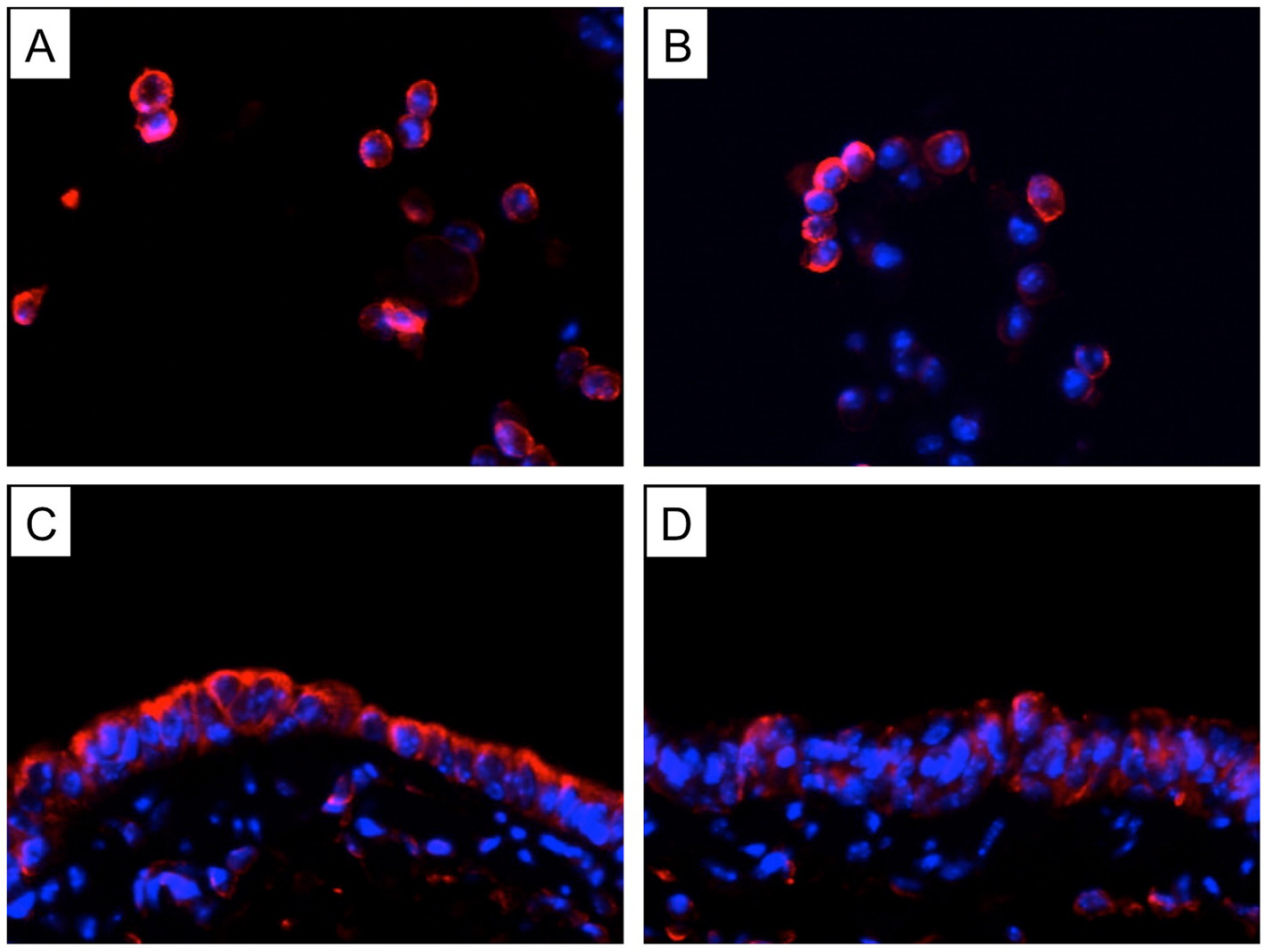
**Cigarette smoke (CS) exposure prior to influenza virus infection results in reduced BAFF expression.** Mice were exposed to room air (RA) or CS for 3 months and then infected with influenza virus or vehicle control. They were sacrificed and evaluated on day 7 after infection. Representative immunohistofluorescence stained lung sections from **(A)** RA-exposed mice with influenza virus infection (cells in bronchial lumen), **(B)** CS-exposed mice with influenza virus infection (cells in bronchial lumen), **(C)** RA-exposed mice with influenza virus infection (bronchial epithelium) and **(D)** CS-exposed mice with influenza virus infection (bronchial epithelium) are shown (BAFF: red, nuclear stain DAPI: blue). Original magnification, ×200.

To study the effects of prior CS exposure on BAFF expression and mucosal IgA responses in the lung during influenza virus infection, mice were infected following 3-month CS exposure and sacrificed on day 7 after infection. Histological examination showed that influenza caused alveolar and airway inflammatory responses characterized by the infiltration of inflammatory cells such as macrophages, neutrophils and lymphocytes, and these responses were exaggerated in mice with prior CS exposure (Figure 3). KC expression was also increased significantly in these mice compared with those only exposed to virus (Figure 4A). Similar changes were found in the total leukocyte counts in BAL fluid (Figure 4B). Importantly, BAFF was reduced significantly in mice with dual exposure of CS and virus compared with mice only exposed to virus (Figure 4C). Immunohistofluorescence staining showed that BAFF was strongly expressed in macrophages and bronchial epithelial cells during infection, and was markedly reduced by the prior CS exposure (Figure 5). Furthermore, the influenza-specific S-IgA levels were also reduced significantly in mice with dual exposure (Figure 4D). In addition, we also measured APRIL mRNA expression in the lung and found that there were no significant differences in APRIL between these two groups (data no shown).

### CS exposure prior to influenza virus infection causes decreased Aicda expression in lung B cells

**Figure 6:**
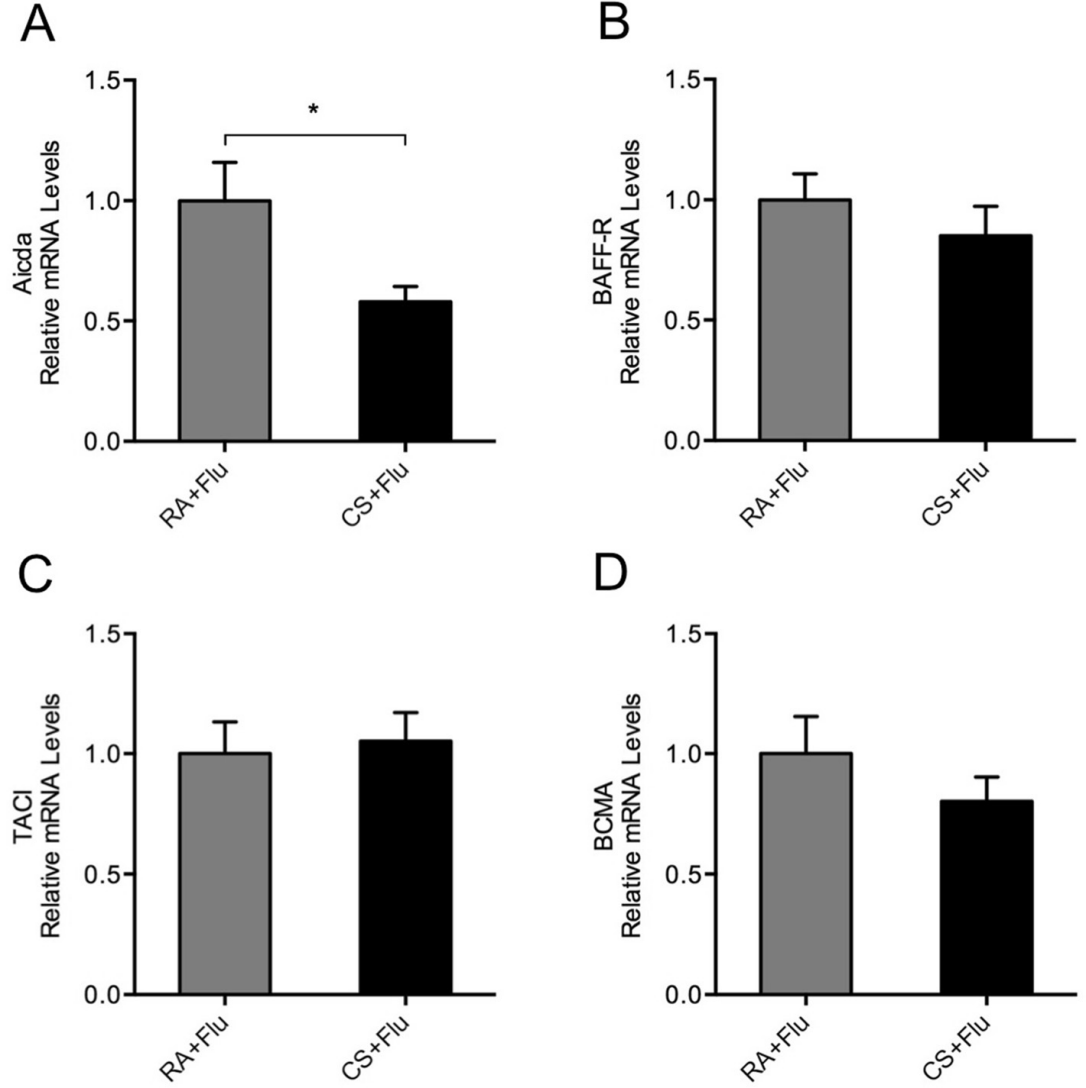
**Cigarette smoke (CS) exposure prior to influenza infection causes decreased Aicda in lung B cells.** Mice were exposed to room air (RA) or CS for 3 months and then infected with influenza virus or vehicle control. Lung B cells were isolated on day 7 after infection. The relative mRNA levels of **(A)** Aicda, **(B)** BAFF receptor (BAFF-R), **(C)** transmembrane activator and calcium modulator cyclophilin ligand interactor (TACI) and **(D)** B cell maturation antigen (BCMA) in lung B cells are shown (n = 5 mice/group). RA + Flu, RA-exposed mice with influenza virus infection; CS + Flu, CS-exposed mice with influenza virus infection; *P < 0.05.

To investigate the effects of prior CS exposure on IgA class switching in lung B cells during influenza virus infection, mice were infected following 3-month CS exposure and lung B cells were isolated on day 7. We found that Aicda (the hallmark of B cells undergoing active class switching) mRNA expression was significantly decreased in lung B cells from the infected mice with prior CS exposure compared with mice only exposed to virus (Figure 6A), while there were no significant changes in the mRNA expression of BAFF-R, TACI and BCMA in lung B cells between these two groups (Figure 6B, C and D).

### BAFF neutralization results in reduced IgA responses in the lung during influenza virus infection

**Figure 7:**
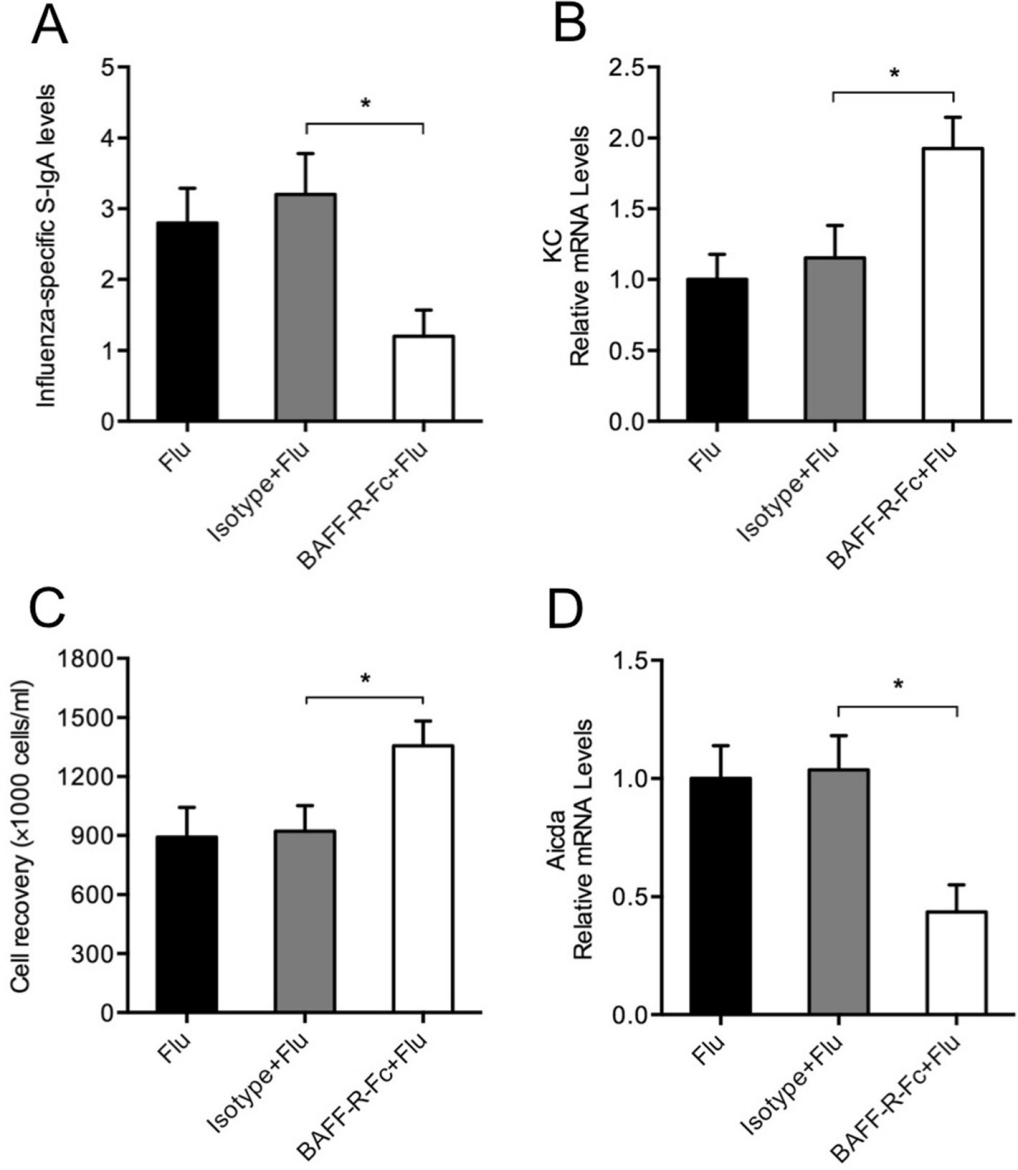
**BAFF neutralization results in reduced IgA responses in the lung during influenza virus infection.** Mice were intranasally administered with BAFF-R-Fc, isotype control or PBS and infected with influenza virus. They were sacrificed and evaluated on day 7 after infection. **(A)** The influenza-specific secretory IgA (S-IgA) levels in bronchoalveolar lavage (BAL) fluid, **(B)** the relative mRNA levels of keratinocyte-derived chemokine (KC) in the lung, **(C)** the total leukocyte counts in BAL fluid and **(D)** the relative mRNA levels of Aicda in lung B cells are shown (n = 5 mice/group). Flu, infected mice with treatment of PBS; Isotype + Flu, infected mice with treatment of isotype control; BAFF-R-Fc + Flu, infected mice with treatment of BAFF-R-Fc; *P < 0.05.

To explore the effect of BAFF inhibition on mucosal IgA responses in the lung during influenza virus infection, mice were intranasally administered with BAFF-R-Fc (the BAFF specific inhibitor) and sacrificed on day 7 after infection. We found that the influenza-specific S-IgA levels were reduced significantly in the lung of BAFF-R-Fc-treated mice compared with isotype control-treated mice (Figure 7A), while KC expression in the lung and total leukocyte counts in BAL fluid were increased significantly in BAFF-neutralized mice (Figure 7B and C). In addition, the Aicda mRNA expression was decreased significantly in lung B cells from BAFF-neutralized mice (Figure 7D).

### Antioxidant NAC prevents the inhibition of virus-mediated BAFF induction by CSE in bronchial epithelial cells

**Figure 8:**
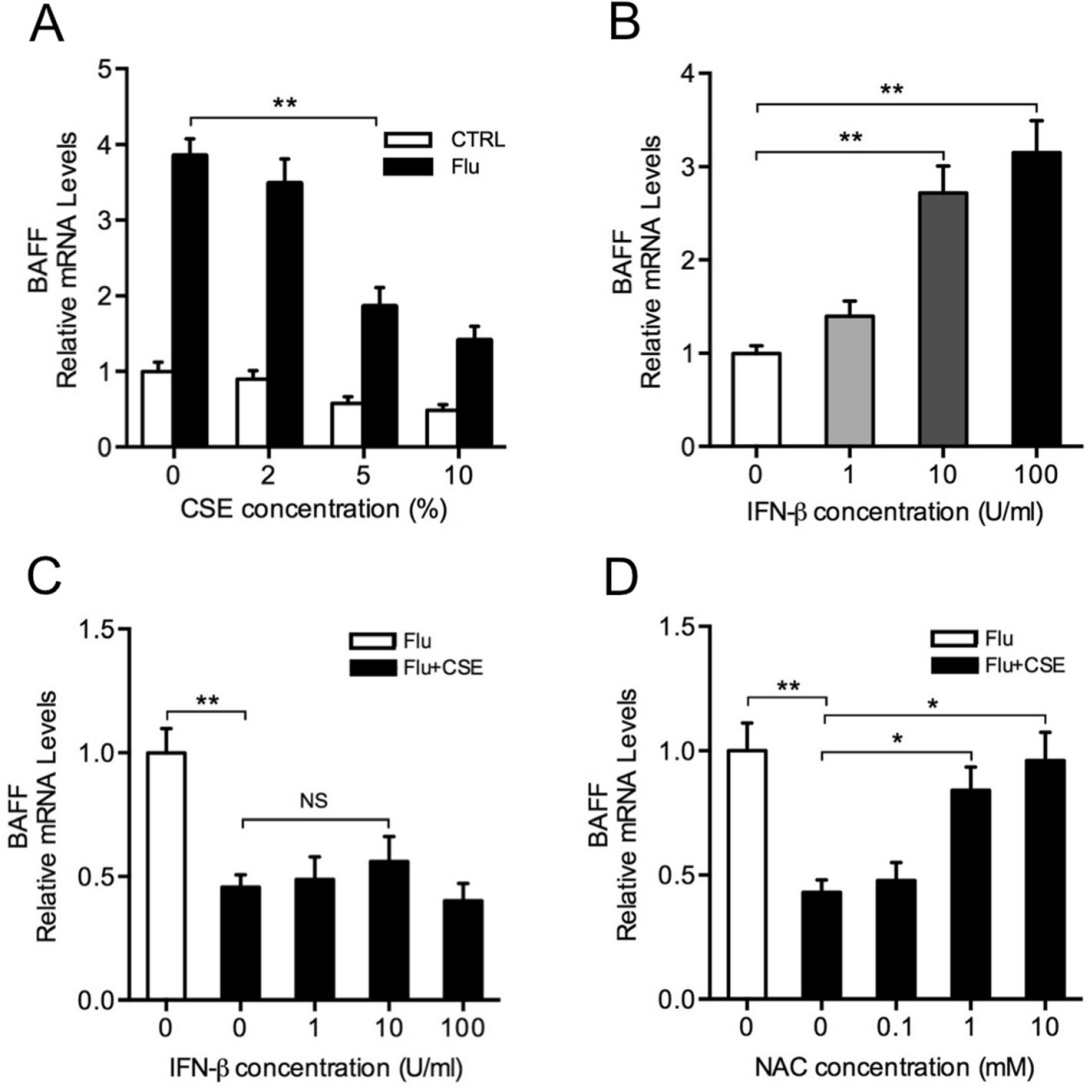
**Antioxidant N-acetylcysteine (NAC) prevents the inhibition of virus-mediated BAFF induction by cigarette smoke extract (CSE). (A)** BEAS-2B cells were treated with indicated concentrations of CSE and/or infected with influenza virus at a multiplicity of infection (MOI) of 0.5 for 24 hours. **(B)** BEAS-2B cells were treated with indicated concentrations of interferon beta (IFN-β) for 24 hours. **(C)** BEAS-2B cells were treated with indicated concentrations of IFN-β in the combination with 5% CSE and influenza virus at 0.5 MOI for 24 hours. **(D)** BEAS-2B cells were pretreated with indicated concentrations of N-acetylcysteine (NAC) for 2 hours and then treated with 5% CSE and influenza virus at 0.5 MOI for 24 hours. The relative mRNA levels of BAFF in BEAS-2B cells are shown. CTRL, without influenza virus infection; Flu, with influenza virus infection; Flu + CSE, with CSE treatment and influenza virus infection; NS, nonsignificant; *P < 0.05; **P < 0.01.

To study the possible mechanisms involved in BAFF inhibition by CS, BEAS-2B cells were cultured and treated with CSE and/or influenza virus as an in vitro model. We found that BAFF was increased significantly after infection, while the virus-induced BAFF expression was reduced by CSE in a dose-dependent manner (Figure 8A). Previous in vitro studies suggest that airway epithelial cells produce BAFF by an IFN-β-dependent mechanism [20] and CSE inhibits virus-induced IFN-β in these cells [21]. To determine whether CSE reduces virus-induced BAFF via IFN-β inhibition, cells were treated with IFN-β alone or in the combination with CSE and virus. We found that BAFF was induced by IFN-β alone in a dose-dependent manner (Figure 8B), while IFN-β treatment did not rescue virus-mediated BAFF induction in the presence of CSE (Figure 8C). However, the inhibition of virus-mediated BAFF induction by CSE was prevented by the pretreatment with the antioxidant NAC in a dose-dependent manner (Figure 8D). In addition, we also measured the viral RNA levels in the influenza virus-treated cells using quantitative PCR and found that the treatments (CSE, IFN-β and NAC) did not significantly affect the replication of influenza virus in these cells (Additional file 1: Figure S1A, B and C).

## Discussion

BAFF has been extensively studied in autoimmune diseases such as systemic lupus erythematosus and rheumatoid arthritis [22], while its role in smoking-related diseases such as COPD is not fully understood. In the present study, we show that CS not only induces the lung inflammation, which plays a central role in COPD [23], but also inhibits BAFF expression in the lung, particularly after long-term exposure. However, previous studies have shown that BAFF expression is increased in the lung of smokers with stable COPD [24,25]. A possible explanation is the recent asymptomatic infection or colonization (or low-grade infection) of respiratory tract with certain bacteria or viruses that may induce BAFF expression. For instance, previous studies have suggested that RSV RNA can be detected in many patients with COPD in the stable state [26]. Although the definite mechanisms for sustained high level of BAFF remain unknown, this dysregulation of BAFF expression in the lung may also contribute to COPD.

Previous studies have demonstrated that BAFF knockout mice exhibit impaired TI antibody responses [11,12], while transient overexpression of BAFF enhances specific immune responses to bacterial vaccines [27]. Recent studies have also shown that BAFF is increased in the lung and associated with the class-switched antiviral IgA antibody responses during RSV infections [14,28]. Consistent with these clinical findings, we show that BAFF expression is induced rapidly and highly in the lung of mice during influenza virus infection, particularly in the early stage. The local influenza-specific S-IgA levels are also markedly increased. Moreover, the neutralization of BAFF in the lung results in reduced S-IgA levels during infection. These results suggest that BAFF promotes the mucosal S-IgA responses in the lung during influenza virus infection. Interestingly, although BAFF expression is increased in the lung of COPD patients [24,25], the S-IgA levels are reduced in their BAL fluid [6]. Recent studies show that IgA accumulates in subepithelial areas of COPD airways due to the impaired transepithelial transport [25,29], which may help to explain the reduced S-IgA levels in BAL fluid from patients with COPD.

In the present study, we have found that CS exposure prior to influenza virus infection results in reduced BAFF expression and local influenza-specific S-IgA levels in the lung. Aicda, a key enzyme essential for IgA class switching and the hallmark of B cells undergoing active class switching [30,31], is also decreased in lung B cells from the virus-infected mice with prior CS exposure compared with those without prior CS exposrue. There are no significant differences in APRIL expression between these two groups. Importantly, the neutralization of BAFF in the lung results in reduced S-IgA levels and Aicda expression during influenza virus infection. Taken together, although prior CS exposure does not alter the expression of the three BAFF receptors on B cells during infection, given the important role of BAFF in inducing Aicda expression and TI IgA class switching in lung B cells in the early stage of infection, these results suggest that CS may hinder early mucosal S-IgA responses through BAFF inhibition in the lung during influenza virus infection.

Previous studies have shown that BAFF can be induced by viral infections in several cell types including airway epithelial cells [14]. Here we show that BAFF is strongly expressed in bronchial epithelial cells during influenza virus infection, suggesting the important role of bronchial epithelial cells in the antiviral immune responses. However, BAFF is markedly reduced in these cells by prior CS exposure. Similarly, our in vitro studies also show that CSE inhibits virus-induced BAFF expression in bronchial epithelial cells. Numerous studies have suggested that CS impairs airway epithelial cell function and oxidative stress is an important mechanism for this impairment [32,33]. Here we demonstrate that the inhibition of virus-induced BAFF expression by CSE can be prevented by the antioxidant NAC. These results suggest that CS may inhibit virus-induced BAFF expression in the lung through oxidative stress.

A number of studies have suggested that CS enhances virus-induced inflammatory responses in the lung as observed in COPD viral exacerbations [34-36]. In the present study, we have also found that CS exposure prior to influenza virus infection results in augmented lung inflammation. However, the underlying mechanisms responsible for this enhanced pulmonary inflammation have not been adequately defined.

It has been suggested that S-IgA, in cooperation with nonspecific innate factors such as mucociliary clearance, provides an efficient defense against external agents without inducing a potentially deleterious inflammatory response [3]. In addition, although S-IgA is classically known for neutralizing toxins, bacteria and viruses at mucosal surfaces, recent studies have suggested that it also has a powerful anti-inflammatory effect due to its ability to interact with DCs through lectin-like receptors [37,38]. This anti-inflammatory effect of S-IgA plays a crucial role in the physiology and in the prevention of tissue damage in many inflammatory diseases [38]. In the present study, we have found that the local mucosal influenza-specific S-IgA levels are decreased significantly in the lung of mice with prior CS exposure during influenza virus infection. These results suggest that the decrease of S-IgA in the lung may also contribute to the exaggerated pulmonary inflammation during viral infection following CS exposure.

In summary, we report that CS inhibits BAFF in the lung, particularly after long-term exposure; BAFF and S-IgA levels are increased during influenza virus infection; CS exposure prior to influenza virus infection results in reduced BAFF and S-IgA in the lung, decreased Aicda in lung B cells as well as augmented pulmonary inflammation on day 7 after infection; BAFF neutralization results in reduced S-IgA levels and Aicda expression during infection; CSE inhibits virus-mediated BAFF induction in bronchial epithelial cells in vitro, while this inhibition can be prevented by the antioxidant NAC. Our findings indicate that CS may hinder early mucosal IgA responses in the lung during influenza virus infection through oxidative inhibition of BAFF, which might contribute to the increased incidence and severity of viral infections in smokers. We believe that a comprehensive understanding of how CS exposure affects lung mucosal immune responses to respiratory infections will provide new insight into development and progression of COPD.

## Additional file

Additional file 1:
**Figure S1.** The viral RNA levels in influenza virus-infected BEAS-2B cells with different treatments.

## Abbreviations

Aicda: Activation-induced cytidine deaminase
APRIL: A proliferation-inducing ligand
BAFF: B cell activating factor belonging to the tumor necrosis factor family
BAFF-R: BAFF receptor
BAFF-R-Fc: BAFF-R Fc fusion protein
BAL: bronchoalveolar lavage
BCMA: B cell maturation antigen
COPD: Chronic obstructive pulmonary disease
CS: Cigarette smoke
CSE: CS extract
DCs: Dendritic cells
ELISA: Enzyme-linked immunosorbent assay
IFN-β: Interferon beta
KC: Keratinocyte-derived chemokine
NAC: N-acetylcysteine
PBS: Phosphate buffered saline
PCR: Polymerase chain reaction
RA: Room air
RSV: Respiratory syncytial virus
S-IgA: Secretory immunoglobulin A
TACI: Transmembrane activator and calcium modulator cyclophilin ligand interactor
TD: T cell-dependent
TI: T cell-independent
