# Supplementary material for: Cigarette smoke inhibits BAFF expression and mucosal immunoglobulin A responses in the lung during influenza virus infection
Source: Respir Res. 2015 Mar 14;16(1):37. doi: 10.1186/s12931-015-0201-y (PMC4364338; doi:10.1186/s12931-015-0201-y)
Supplement: Additional file 1: — Figure S1. The viral RNA levels in influenza virus-infected BEAS-2B cells with different treatments. [file 12931_2015_201_MOESM1_ESM.pdf]

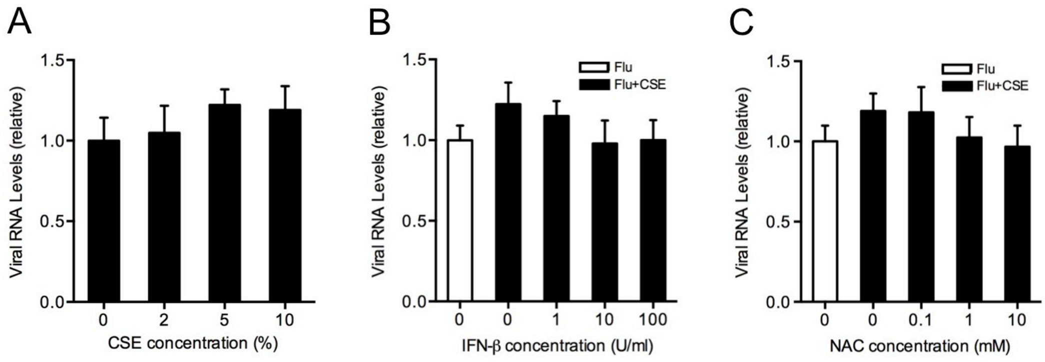


**Figure S1. The viral RNA levels in influenza virus-infected BEAS-2B cells with different treatments.** (A) BEAS-2B cells were treated with indicated concentrations of cigarette smoke extract (CSE) and infected with influenza virus at a multiplicity of infection (MOI) of 0.5 for 24 hours. (B) BEAS-2B cells were treated with indicated concentrations of interferon beta (IFN-β) in the combination with 5% CSE and influenza virus at 0.5 MOI for 24 hours. (C) BEAS-2B cells were pretreated with indicated concentrations of N-acetylcysteine (NAC) for 2 hours and then treated with 5% CSE and influenza virus at 0.5 MOI for 24 hours. The viral RNA levels of influenza virus in BEAS-2B cells are shown. Flu, with influenza virus infection; Flu+CSE, with CSE treatment and influenza virus infection.
